# Process Optimization for Improved Phenolic Compounds Recovery from Walnut (*Juglans regia* L.) Septum: Phytochemical Profile and Biological Activities

**DOI:** 10.3390/molecules23112814

**Published:** 2018-10-30

**Authors:** Marius Emil Rusu, Ana-Maria Gheldiu, Andrei Mocan, Cadmiel Moldovan, Daniela-Saveta Popa, Ioan Tomuta, Laurian Vlase

**Affiliations:** 1 Department of Pharmaceutical Technology and Biopharmaceutics, Faculty of Pharmacy, “Iuliu Hatieganu” University of Medicine and Pharmacy, 8 Victor Babes, 400012 Cluj-Napoca, Romania; marius.e.rusu@gmail.com (M.E.R.); tomutaioan@umfcluj.ro (I.T.); laurian.vlase@umfcluj.ro (L.V.); 2 Department of Pharmaceutical Botany, Faculty of Pharmacy, “Iuliu Hatieganu” University of Medicine and Pharmacy, 8 Victor Babes, 400012 Cluj-Napoca, Romania; Gheldiu.Ana@umfcluj.ro (A.-M.G.); moldovan.cadmiel@yahoo.com (C.M.); 3 Department of Toxicology, Faculty of Pharmacy, “Iuliu Hatieganu” University of Medicine and Pharmacy, 8 Victor Babes, 400012 Cluj-Napoca, Romania

**Keywords:** walnut septum, polyphenols, phytosterols, HPLC-MS/MS, Ultra-Turrax extraction, biological activity, antioxidant activity, experimental design, optimization, phytochemicals

## Abstract

Plant by-products can be valuable sources of polyphenol bioactive compounds. Walnut (*Juglans regia* L.) is a very important tree nut rich in biologically active molecules, but its septum was scarcely researched. Experimental data indicated a hypoglycemic effect of septum extracts, with almost no details about its phytochemical composition. The main objectives of this study were: (1) to obtain walnut septum (WS) extracts with high content in bioactive compounds and antioxidant activity based on an original experimental design; (2) characterization of the phytochemical profile of the WS extracts using HPLC-MS/MS; (3) evaluation of the biological potential of the richest polyphenolic WS extract. The variables of the experimental design were: extraction method (maceration and Ultra-Turrax extraction), temperature, solvent (acetone and ethanol), and percentage of water in the solvent. The first quantifiable responses were: total phenolic content, total flavonoid content, condensed tannins, and ABTS antioxidant capacity. The phytochemical profile of lyophilized extracts obtained by Ultra-Turrax extraction (UTE), the most efficient method, was further determined by HPLC-MS/MS analysis of individual polyphenolic and phytosterols compounds. It is the first study to assay the detailed composition of WS in hydrophilic and lipophilic compounds. The biological potential of the richest polyphenolic WS extract was also evaluated by FRAP and DPPH antioxidant capacity and the inhibition of tyrosinase, an enzyme involved in the browning in fruits and vegetables, skin wrinkles and aging. Conclusion: The phytochemical profile of the analyzed extracts proves that WS can be a valuable source of biologically active compounds (polyphenols) for food and/or pharmaceutical industry and warrant the continuation of current research in further evaluating its bioactive potential.

## 1. Introduction

Each year the food industry creates a substantial amount of waste and serious issues are associated with its disposal. Coupled with the tendency of the consumers to avoid foods prepared with chemical origin preservatives, many studies have been recently conducted, intended to find natural alternatives, such as plant by-products, rich in bioactive compounds with high potential for health and pharmaceutical industry [1,2].

In the last decades, the number of people with body mass problems increased in the world obesogenic culture. Overweight and obesity are increasingly seen as major concerns for human health [3]. Processed food, the so called “junk food”, with high content of carbohydrates, fats, and salt, is linked to overweight and obesity via several mechanisms [4]. Excessive body weight, associated with several pro-inflammatory cytokines (e.g., leptin, interleukin 6, interleukin 8, tumor necrosis factor-alpha), and a chronic, low-grade inflammation [5], is seen as a major risk factor for obesity-associated diseases, such as metabolic dysfunction [6], diabetes [7], cardiovascular diseases [8], and cancers [9], including endometrial [10], breast [11], gastrointestinal [12], pancreatic [13], prostate [14], hepatic [15], renal [16], colorectal [17].

Epidemiological studies and clinical trials demonstrated that diets with high intake of plant origin foods (vegetables, fruits, nuts) can safeguard against excessive weight-related diseases and offer powerful protection for the cardiovascular, gastrointestinal, and immune systems [18,19]. Phytochemicals, including carotenoids, glucosinolates, and polyphenols, work synergistically to reduce inflammation and oxidation, providing defense against initiation and evolution of ailments [20]. Phenolic acids and flavonoids, the major contributors of the polyphenols group, act as natural antioxidants decreasing the risk of degenerative diseases [21]. Polyphenols are compounds which donate electrons or hydrogen atoms to reactive radicals preventing the degradation of vital molecules or cellular damage [22]. Besides their role as antioxidants in the detoxifying system with a scavenging role against reactive oxygen or nitrogen species, plant polyphenols can take part in the enzymatic pathways involved in the energetic balance or act as signaling molecules in the cell [23]. In addition to the antioxidant activity, several studies [24,25] confirmed the antimicrobial activity of the polyphenols, making them a good substitute to antibiotics and chemical preservatives. 

Walnut (*Juglans regia* L.), a valued crop of high economic importance, represents a good source of nutritional and nutraceutical compounds [26]. Besides the well-known antioxidant, antibacterial, and anti-inflammatory bioactivity of the walnut kernel [27], several studies proved that walnut leaves [28] and green husk [29] could induce the same great health benefits. Walnut membrane septum, another by-product of this valuable plant, was traditionally used as a cold remedy or cough suppressant, presented a hypoglycemic activity in an experimental animal model [30], and improved blood profile in murine experiments [31]. Walnut septum extracts had no acute or subchronic toxicity in rat [32]. However, to the best of our knowledge the phytochemical profile of walnut membrane septum has not been reported in the literature. 

The aim of the study was the determination of phenolic and phytosterol compounds from the walnut septum based on an experimental design. Extraction method, solvent, temperature, and water percentage, the variables of the study, were combined with statistical tools and analysis using LC-MS/MS in order to determine the optimal extraction conditions, identification, and quantification of main phenolic and phytosterol molecules from septum. Several methods were employed to determine the antioxidant capacity (ABTS, DPPH, and FRAP) and the enzymatic inhibitory activity. 

## 2. Materials and Methods

### 2.1. Chemicals

The reagents used in this study were: vanillin (99%), sodium carbonate, ferric chloride, 6-hydroxy-2,5,7,8-tetramethylchromane-2-carboxylic acid (Trolox) (97%), diammonium 2,2′-azino- bis(3-ethylbenzothiazoline-6-sulfonate) (ABTS) (>98%), 2,2-diphenyl-1-(2,4,6-trinitro-phenyl) hydrazine (DPPH), 2,4,6-Tris(2-pyridyl)-*S*-triazine (TPTZ) (≥99%), dimethyl sulfoxide (DMSO) (≥99%), phosphate buffer, mushroom tyrosinase, 3,4-Dihydroxy-l-phenylalanine (l-DOPA) (≥98%), and kojic acid were purchased from Sigma (Sigma Aldrich Chemie GmbH, Schnelldorf, Germany). Folin–Ciocâlteu reagent, hydrochloric acid (37%), acetone, ethanol, methanol were purchased from Merck (Darmstadt, Germany). Aluminum chloride (≥98%) was purchased from Carl Roth (Karlsruhe, Germany). All reagents were of analytical grade and all solvents were of LC grade. Water was of Milli-Q-quality.

The standards used for both spectrophotometric and LC-MS/MS analysis were: quercetin (≥95%), hyperoside (quercetin 3-d-galactoside) (≥97%), isoquercitrin (quercetin 3-β-d-glucoside) (≥98%), quercitrin (quercetin 3-rhamonoside) (≥78%), (+)-catechin (≥96%), (−)-epicatechin (≥90%), vanillic acid (≥97%), syringic acid (≥95%), protocatechuic acid (3,4-dihydroxybenzoic acid) (≥97%), campesterol (~65%), ergosterol (≥95%), and stigmasterol (~95%) purchased from Sigma-Aldrich, gallic acid (≥98%) purchased from Merck (Darmstadt, Germany), and beta-sitosterol (≥80%) purchased from Carl Roth (Karlsruhe, Germany). 

### 2.2. Plant Samples

Walnuts (*Juglans regia* L.) of high quality were provided by an organic orchard in Bucium, Maramureş County, Romania. In the autumn of 2016, walnuts were harvested and kept in a dark, airy shelter, at temperatures ~0 °C. At the beginning of March 2017, the unshelled walnuts were delivered to the Faculty of Pharmacy, “Iuliu Hatieganu” University of Medicine and Pharmacy Cluj-Napoca, Romania, and identified by Dr. Andrei Mocan from the Department of Pharmaceutical Botany. A voucher specimen was deposited in the Herbarium of this Department. The unshelled walnuts were cracked and the walnut septum (WS) removed from the hard shells just prior to the extractions.

### 2.3. Samples

WS was ground in a coffee grinder (Argis, RC-21, Electroarges SA, Curtea de Argeș, Romania) for 5 min. Then, the ground septum powder was screened through a 200 µm Retsch sieve.

### 2.4. Preparation of Extracts

The extraction process was carried out based on a D-optimal experimental design developed by Modde software, version 11.0 (Sartorius Stedim Data Analytics AB, Umeå, Sweden) using four variable factors: preparation method, temperature, solvent, and percentage of water in solvent (Table 1).

WS was weighed (2 g) and mixed with the extraction solvent (20 mL) in Falcon tubes. The Ultra-Turrax extraction (UTE) was performed in two steps: using an Ultra-Turrax homogenizer (T 18; IKA Labortechnik, Staufen, Germany) for 2 min (1 min at 9500 rpm and 1 min at 13,500 rpm) [33] and again 2 min using a Vortex RX-3 (Velp Scientifica, Usmate, Italy). The homogenate was centrifuged (Hettich, Micro 22R, Andreas Hettich GmbH & Co., Tuttlingen, Germany) 15 min at 3000 rpm, maintaining the extraction temperature. The supernatant was carefully separated, and the solvent removed under vacuum at 40 °C using a rotary evaporator (Hei-VAP, Heidolph Instruments GmbH & Co., Schwabach, Germany). The dry residue was taken up in water, placed in amber glass vials, and lyophilized (Advantage 2.0, SP Scientific, Warminster, PA, USA).

For the maceration method, WS (2 g) was added to Erlenmayer flasks with the extraction solvent and kept for 10 days at 20, 30, and 40 °C (Conterm Oven, JP Selecta S.A., Barcelona, Spain) and stirred twice daily. After 10 days, the samples were centrifuged (Hettlich Micro 22R, Andreas Hettlich GmbH & Co. KG, Tuttlingen, Germany) 10 min at 5300 rpm, maintaining the extraction temperature. Then, the supernatant was separated, the solvent evaporated and the remaining water removed as seen before. After lyophilization, the samples (for both extraction methods) were stored at room temperature.

For further determinations, lyophilized extract was dissolved in EtOH 70% (10 mg/mL). All assays were executed in triplicate.

### 2.5. Quantitative Determinations of Total Bioactive Compounds

#### 2.5.1. Total Phenolic Content

The total phenolic content (TPC) of the WS extracts was determined by Folin-Ciocâlteu spectrophotometric method according to a method described previously [34]. In brief, in a 96 well plate, 20 µL of each sample (WS extracts diluted 5 times) were mixed with 100 µL of FC reagent (diluted 1:10). After 3 min, 80 µL of sodium carbonate solution (7.5% *w/v*) was added to the wells. The plate was incubated for 30 min in the dark at room temperature. A Synergy HT Multi-Detection Microplate Reader with 96 well plates (BioTek Instruments, Inc., Winooski, VT, USA) was used to measure the absorbance at 760 nm against a solvent blank. Gallic acid was used as a reference standard, and the content of phenolics was expressed as gallic acid equivalents (GAE) per dry weight of septum (mg GAE/g dw).

#### 2.5.2. Total Flavonoid Content

The total flavonoid content (TFC) of the WS extracts was determined according to a method described previously [35]. In a 96 well plate, 100 µL of sample extracts were added to 100 µL of 2% AlCl_3_ aqueous solution. The plate was incubated for 15 min in the dark at room temperature. The absorbance at 420 nm was measured against a solvent blank. The TFC was expressed as quercetin equivalents (QE) per dry weight (dw) of vegetal material (mg QE/g dw).

#### 2.5.3. Condensed Tannin Content

The condensed tannin content (CTC) in WS extracts was determined according to a modified version of the vanillin assay described before [36,37]. Briefly, in a 96 well plate, 50 µL of sample WS extracts were added to 250 µL 0.5% vanillin in 4% concentrated HCl in methanol. The plate was incubated for 20 min in the dark at 30 °C. The absorbance at 500 nm was measured against a solvent blank. The condensed tannins were expressed as catechin equivalents (CE) per dry weight (dw) of vegetal material (mg CE/g dw). 

### 2.6. Phytochemical Analysis by LC-MS/MS

The phytochemical profile of lyophilized WS extracts obtained by UTE method was assessed by liquid chromatography coupled with mass spectrometry in tandem (LC-MS/MS). The experiment was carried out using an Agilent 1100 HPLC Series system (Agilent, Santa Clara, CA, USA) equipped with degasser, binary gradient pump, column thermostat, auto sampler, and UV detector. The HPLC system was coupled with an Agilent Ion Trap 1100 SL mass spectrometer (LC/MSD Ion Trap VL).

#### 2.6.1. Identification and Quantification of Polyphenolic Compounds

A previously LC-MS/MS method [38,39,40,41] was slightly modified (replacing of sodium phosphate with acetic acid in the mobile phase) and applied for the identification of 18 polyphenols in the sample WS extracts: caftaric acid, gentisic acid, caffeic acid, chlorogenic acid, *p*-coumaric acid, ferulic acid, sinapic acid, hyperoside, isoquercitrin, rutozid, myricetol, fisetin, quercitrin, quercetin, patuletin, luteolin, kaempferol, and apigenin. In brief, chromatographic separation was performed on a reverse-phase analytical column (Zorbax SB-C18, 100 mm × 3.0 mm i.d., 3.5 µm) with a mixture of methanol: 0.1% acetic acid (*v*/*v*) as mobile phase and a binary gradient. The elution started with a linear gradient, beginning with 5% methanol and ending at 42% methanol at 35 min; isocratic elution followed for the next 3 min with 42% methanol; rebalancing in the next 7 min with 5% methanol. The flow rate was 1 mL/min, the column temperature 48 °C and the injection volume was 5 μL.

The detection of the compounds was performed on both UV and MS mode. The UV detector was set at 330 nm until 17 min (for the detection of polyphenolic acids, then at 370 nm until 38 min to detect flavonoids and their aglycones. The MS system operated using an electrospray ion source in negative mode (capillary +3000 V, nebulizer 60 psi (nitrogen), dry gas nitrogen at 12 L/min, dry gas temperature 360 °C). The chromatographic data were processed using ChemStation and DataAnalysis software from Agilent, USA.

Another LC-MS method was used to identify other six polyphenols in WS extracts: epicatechin, catechin, syringic acid, gallic acid, protocatechuic acid, and vanillic acid. The chromatographic separation was performed on the same analytical column as mentioned before (Zorbax SB-C18, 100 mm × 3.0 mm i.d., 3.5 µm) with a mixture of methanol: 0.1% acetic acid (*v*/*v*) as mobile phase and a binary gradient (start: 3% methanol; at 3 min: 8% methanol; at 8.5 min: 20% methanol; keep 20% methanol until 10 min then rebalance column with 3% methanol). The flow rate was 1 mL/min and the injection volume was 5 μL. The detection of the compounds was performed on MS mode (Table 2). The MS system operated using an electrospray ion source in negative mode (capillary +3000 V, nebulizer 60 psi (nitrogen), dry gas nitrogen at 12 L/min, dry gas temperature 360 °C). All identified polyphenols were quantified both in the WS extracts and hydrolyzed WS extracts (equal quantities of extract and 4 M HCl kept 30 min on 100 °C water bath) on the basis of their peak areas and comparison with a calibration curve of their corresponding standards (epicatechin, catechin, syringic acid, gallic acid, protocatechuic acid, vanillic acid, hyperoside, isoquercitrin, quercitrin). The results were expressed as milligrams of phenolic per gram of dry weight of septum extract.

#### 2.6.2. Identification and Quantification of Phytosterols

The pytosterols in the septum extracts were determined according to a method described previously [42,43]. In brief, chromatographic separation was performed on a Zorbax SB-C18 (100 mm × 3.0 mm i.d., 5 µm) column (Agilent Technologies) with a mixture of methanol:acetonitrile (10:90, *v*/*v*) and isocratic elution, at 45 °C with a flow rate of 1 mL/min. The detection of analytes was performed in the multiple reaction monitoring (MRM) mode for the quantification of phytosterols, positive ion detection, using an ion trap mass spectrometer equipped with an atmospheric pressure chemical ionization (APCI) source (capillary −4000 V, nebulizer 60 psi (nitrogen), vaporizer 400 °C, dry gas nitrogen at 7 L/min, dry gas temperature 325 °C).

Four external standards were used for quantification: beta-sitosterol, stigmasterol, campesterol, and ergosterol. The identified phytosterols (beta-sitosterol and campesterol) were quantified on the basis of their peak areas and comparison with a calibration curve of their corresponding standards. The results were expressed as milligrams phytosterols per gram of dry weight of septum extract.

### 2.7. Antioxidant Activity Assays

#### 2.7.1. ABTS Radical Cation Scavenging Activity

The antiradical activity of WS extracts was determined according to the trolox equivalent antioxidant capacity (TEAC) assay described previously [35,44]. The scavenging activity against ABTS radical cation (2,2′-azino-bis(3-ethylbenzothiazoline)-6-sulphonic acid) was assessed and used to plot the trolox calibration curve. The total antioxidant activity (TAA) according to TEAC assay was expressed as trolox equivalents (TE) per gram of dry lyophilized extract (mg TE/g dw extract). This assay was used during the screening phase of the study for the evaluation of total antioxidant activity of the 23 samples obtained by either maceration or UTE method.

#### 2.7.2. DPPH Radical Scavenging Activity

The antiradical activity of WS extracts was assessed using a method previously described [45]. The capacity to scavenge the free radical DPPH was determined in a 96 well plate mixing 30 μL of sample solution with a 0.004% methanolic solution of DPPH for 30 min in the dark. The absorbance at 517 nm was measured against a solvent blank. Trolox was used as a reference standard and the results were expressed as trolox equivalents per gram of dry lyophilized extract (mg TE/g dw extract). This assay was performed on the richest polyphenolic WS extract.

#### 2.7.3. FRAP Assay

The reduction capacity of the WS extract was evaluated by FRAP (ferric reducing antioxidant power) assay that analyzes the blue-colored Fe^2+^-TPTZ formed by the reduction of Fe^3+^-TPTZ. A method previously described [46] was used with slight modifications. In brief, 25 μL of sample were incubated with 175 μL FRAP reagent (300 mM acetate buffer, pH 3.6: 10 mM TPTZ in 40 mM HCl: 20 mM FeCl_3_·6H_2_O in 40 mM HCl, 10:1:1, *v*/*v*/*v*) in a 96 well plate for 30 min in the dark. Trolox was used as an external standard (calibration curve obtained for 0.01–0.10 mg/mL) and the absorbance was measured at 593 nm. The results were expressed as trolox equivalents per gram of dry lyophilized extract (mg TE/g dw extract). This assay was done on the richest polyphenolic WS extract.

### 2.8. Tyrosinase Inhibitory Activity

The tyrosinase inhibitory activity of WS extract was evaluated by a 96-well microplate method previously described [47] with slight changes. Briefly, four wells were designated (WS lyophilized extract dissolved in water containing 5% DMSO) as follows: (A) 66 mM phosphate buffer, pH 6.6 (PB) (120 μL) and mushroom tyrosinase in the same buffer, 46 U/mL (MT) (40 μL); (B) only PB (160 μL); (C) PB (80 μL), MT (40 μL) and the sample (40 μL); (D) PB (120 μL) and the sample (40 μL). After 10 min incubation at room temperature, 2.5 mM L-DOPA prepared in PB (40 μL) was added in all wells. The microplate was kept again at room temperature for 20 min and the absorbance was measured at 475 nm. The tyrosinase inhibitory activity was assessed using kojic acid as an external standard (0.01–0.10 mg/mL). The inhibition percentage of enzymatic activity was calculated by the following equation: [(A − B) − (C − D)] × 100/(A − B). The results were expressed as milligram kojic acid equivalents per gram of dry lyophilized extract (mg KAE/g dw extract). This evaluation was carried out for the richest polyphenolic WS extract.

### 2.9. Identification of the Experimental Conditions to Obtain WS Extracts Rich in Phytochemicals

During the screening step, the quantifiable responses TPC, TFC, CTC, TAA according to TEAC assay, were analyzed by the Modde software, version 11.0, to identify the optimal extraction conditions. For the optimization step, individual phenolic and phytosterol levels were evaluated and the independent factors investigated were working temperature, organic solvent, and percentage of water in solvent mixture. The responses were identification and quantification of each quantified phytochemical compound: epicatechin, catechin, syringic acid, gallic acid, protocatechuic acid, vanillic acid, hyperoside, isoquercitrin, quercitrin, campesterol, beta-sitosterol. 

### 2.10. Statistical Analysis

All samples were analyzed in triplicate (*n* = 3) and the results were expressed as the mean ± Standard Deviation (SD).

## 3. Results and Discussion 

### 3.1. Fitting of the Experimental Data with the Models

The independent and dependent variables of experimental design evaluated for WS extraction yield during the screening step are shown in Table 1. The independent variables (factors) were the extraction method, working temperature, organic solvent, and percent of water in solvent mixture. The dependent variables (responses) were TPC, TFC, CTC, and TAA. The matrix of the experimental design generated by the Modde software, version 11.0, along with the responses obtained after performing all the experimental runs are given in Table 3. 

As it can be observed from the results, the extraction yields of TPC, TFC, CTC, as well as the TAA, were influenced by the extraction method and factors evaluated in the experimental design.

For evaluation of the partial least squares regression (PLS) for fitting of the experimental data with the experimental design, R^2^ and Q^2^ were used as statistical parameters. The goodness of fit is overestimated by the value of R^2^, describing the percent of the variation of the response explained by the model, and underestimated by the value of Q^2^, representing the percent of the variation of the response predicted by the model according to cross validation. The two aforementioned statistical parameters are the most reliable for describing the model validity; high values and a difference of no more than 0.2–0.3 between these two indicate a high predictive power of a good model. Furthermore, the reproducibility of the model was evaluated considering the variation of the response under the same experimental conditions (pure error) in comparison with the total variation of the response.

The summary of fit for the responses evaluated in the screening step is presented in Table 4 and the regression coefficients are given in Table 5. 

The experimental setup is appropriate for the purpose of the study and, by working under the same experimental conditions, the replicates generated similar responses, this statement being supported by the reproducibility values > 0.82. The response variation is considered by the developed models (R^2^ > 0.61) and the predictive capacity was found to be adequate (Q^2^ > 0.37). The analysis of variance (ANOVA), shown in Table 4, supports the statistical significance of the model, with *p*-value in the range of 0.001 to 0.028 and *F*-values between 3.26 and 8.29. According to the results given in Table 4, the fitting models were found to be adequate to describe the experimental data, taking into account that the values for the lack of fit were not significant in extent to the pure error (3.60 for TPC, 5.79 for TFC, 1.88 for CTC, and 6.18 for TAA).

The independent and dependent variables of experimental design evaluated for WS extracts during the optimization step are shown in Table 6. The independent factors were temperature, organic solvent, and percentage of water in solvent mixture. The responses were identification and quantification of the following bioactive compounds: epicatechin, catechin, syringic acid, gallic acid, protocatechuic acid, vanillic acid, hyperoside, isoquercitrin, quercitrin, campesterol, beta-sitosterol. 

For the matrix of the experimental design the same Modde software and version was used as in the screening step, and the responses obtained after performing all the experimental runs are given in Table 7. For fitting the experimental data with the experimental design, the same statistical parameters were determined as mentioned previously (R^2^, Q^2^, regression, lack of fit, and pure error) and they are presented in Table 8. By analyzing the results shown in Table 8, the fitting models were adequate to describe the experimental data, considering the values of the reproducibility, lack of fit, and pure error. The regression coefficients for bioactive compounds determined in WS extracts are shown in Table 9.

### 3.2. The Influence of Studied Variables on TPC, TFC, CTC, TAA, and Individual Bioactive Compounds

The different working conditions for walnut septum extracts are shown in Table 3. A number of 13 samples were obtained by UTE method, while 10 samples were obtained by maceration. The working temperature was in the range of 20 to 40 °C, the two solvents used were acetone and ethanol mixed with water in various proportions.

**Figure 1:**
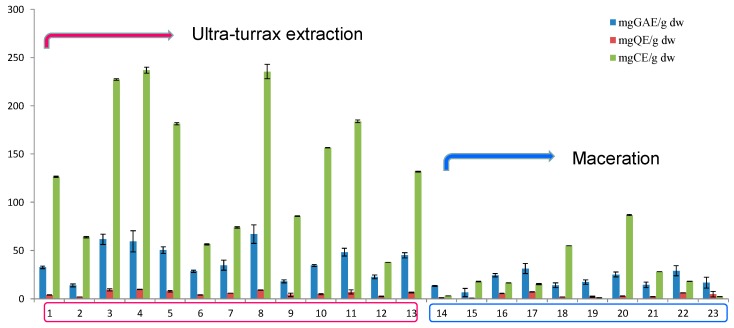
Total phenolic content (gallic acid equivalents, GAE), total flavonoid content (quercetin equivalents, QE), and condensed tannin content (catechin equivalents, CE) of analyzed walnut septum extracts.

**Figure 2:**
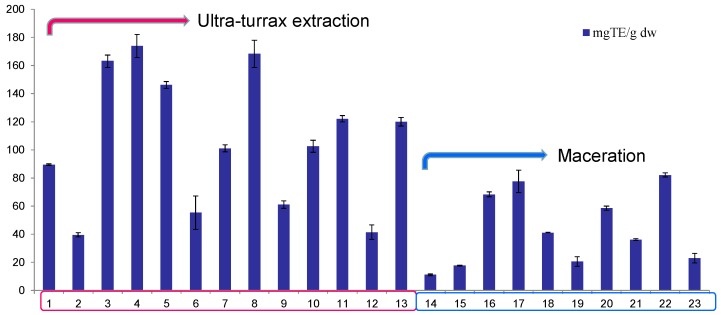
The total antioxidant activity evaluated through ABTS radical cation scavenging activity assay (expressed as Trolox equivalents, TE) of analyzed walnut septum extracts.

The results for TPC, TFC, and CTC for the 23 walnut septum extracts are depicted in Figure 1, and the results for TAA of the same 23 WS extracts are shown in Figure 2. As it can be observed, acetone presents higher extraction power for the bioactive compounds, while the method with higher extraction efficiency was UTE. 

Longer extraction time period and high amount of solvent are involved in maceration. In this case, phenolic compounds may also suffer oxidation, hydrolysis, and ionization of the molecules. These could be reasons for the observed results regarding the two extraction methods that were used. Moreover, the ultrasound energy can leach the bioactive compounds of interest, thus increasing the yield of extraction [48].

There was a good correlation between the content of bioactive compounds from WS extracts and their antioxidant activity. The extracts presenting the highest content of these compounds exhibited the highest TAA and these extracts were obtained by UTE method. Therefore, the 13 extracts obtained by this method were further analyzed in terms of the factors influencing their extraction efficiency.

**Figure 3:**
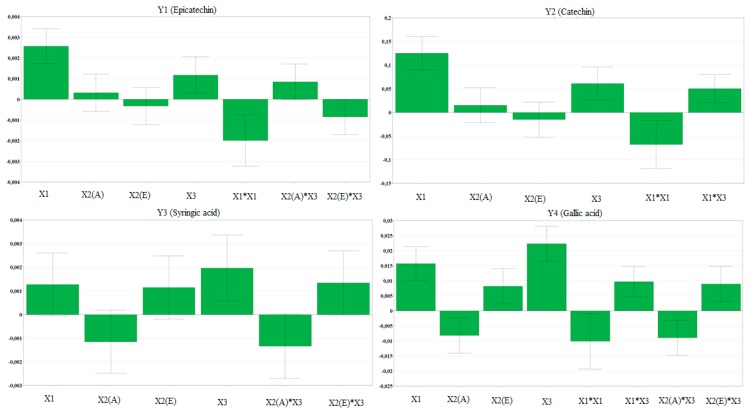

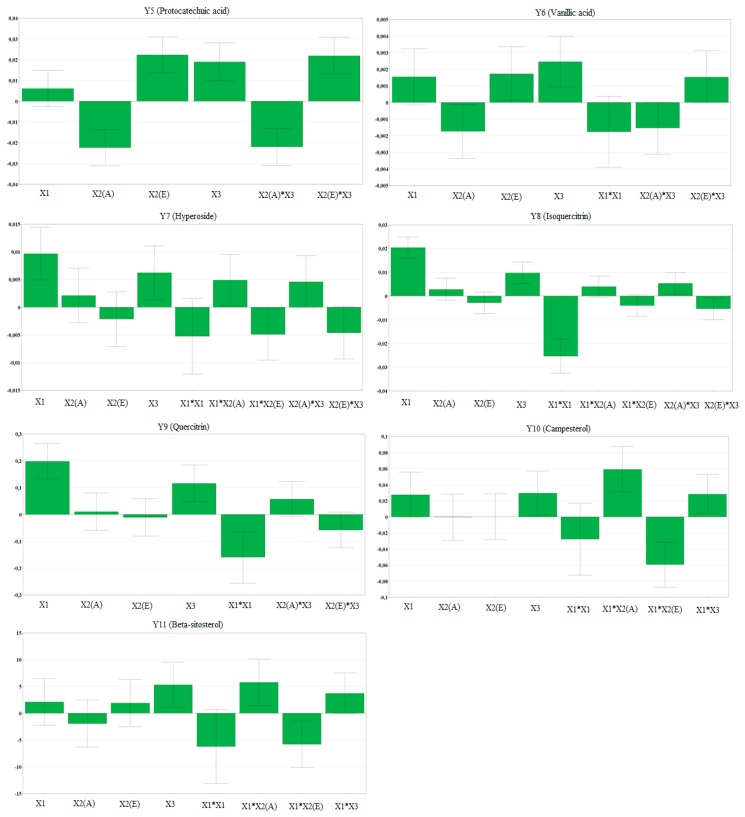
Influence of working conditions on the bioactive compounds recovery from walnut septum extracts, presented as scaled and centered coefficient plots. X_1_—temperature (°C); X_2_(A)—solvent type (acetone), X_2_(E)—solvent type (ethanol); X_3_—water % in mixture with solvent; Y_1_, Y_2_, Y_3_, Y_4_, Y_5_, Y_6_, Y_7_, Y_8_, Y_9_, Y_10_, Y_11_—dependent variables (bioactive compounds) according to Table 5.

**Figure 4:**
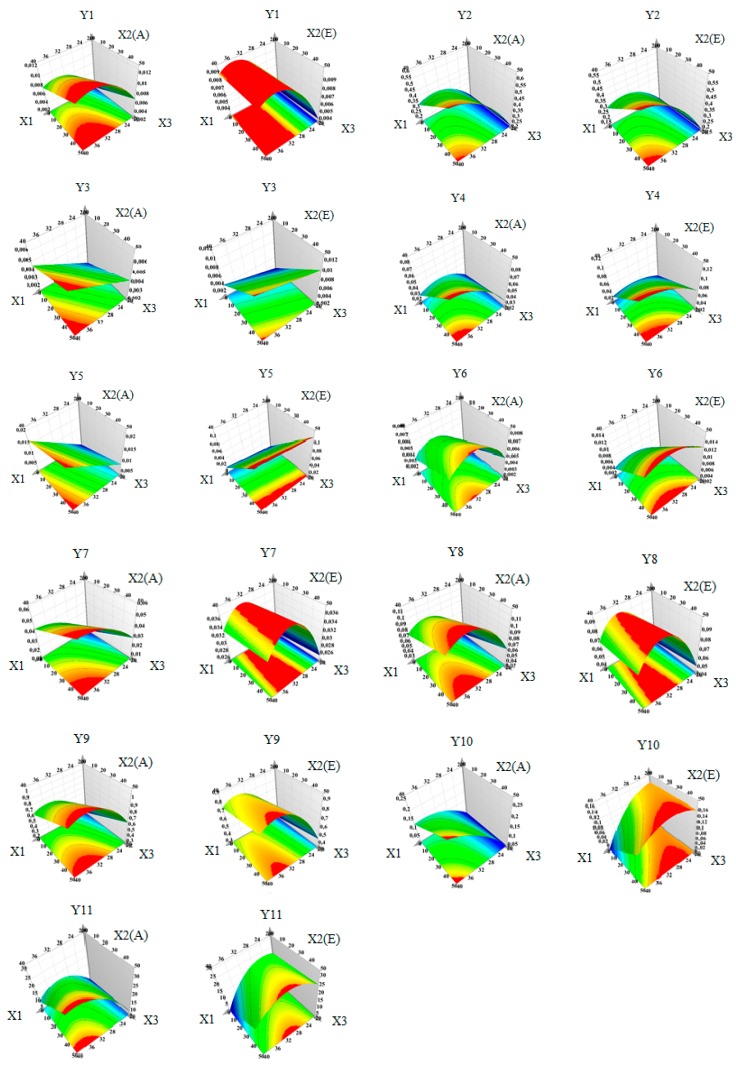
Response surface for predicting the bioactive compounds recovery from walnut septum extracts with respect to: X_1_—temperature (°C); X_2_(A)—solvent type (acetone), X_2_(E)—solvent type (ethanol); X_3_—water % in mixture with solvent (the regions in red represent the domains of working conditions that assure a maximum extraction yield for the evaluated bioactive compounds).

The polyphenols in WS extracts, which were determined and quantified by HPLC/MS, are summarized in Table 8. The compounds found in the highest amount were catechin and quercitrin. The two phytosterols determined in the analyzed samples were campesterol and beta-sitosterol. Forwards, the manner in which the extraction yield of the main bioactive compounds is influenced by the working conditions is briefly presented. Moreover, the influence of working conditions on the bioactive compounds extraction yield from WS samples are presented as scaled and centered coefficient plots in Figure 3. In addition, the response surfaces for predicting the extraction yield of bioactive compounds from WS extracts with respect to the evaluated working conditions are shown in Figure 4.

For epicatechin (Y_1_), the highest extraction yield is obtained when working at high temperature. The high percentage of water in acetone influences its extraction to a lesser extent. For this bioactive compound, the use of ethanol in the extraction mixture is not favorable. Catechin (Y_2_) extraction is influenced by temperature and solvent. More precisely, for this compound the best working conditions would be high temperature and high percentage of water in solvent, according to the response surface generated by Modde software. For syringic acid (Y_3_) extraction, the optimum extraction conditions are high temperature and high amount of water in ethanol as solvent. The extraction of gallic acid (Y_4_) can be optimized if working at high temperature and high amount of water in the solvent mixture. In this case, a higher extraction yield can be obtained if ethanol is used instead of acetone. For protocatechuic acid (Y_5_), all the evaluated factors seemed to have a statistically significant influence upon the extraction power, the most important working parameter being the amount of water in ethanol. Vanillic acid (Y_6_) extraction power increases with the increase of temperature and water percentage in solvent. If acetone is used in mixture with water, then the working temperature does not influence the extraction yield. On the contrary, if a mixture of ethanol with water is used, then an increase in temperature will lead to an increase in the extraction power. Hyperoside (Y_7_) is best extracted in the following working conditions: high temperature, high percentage of water in solvent, and acetone as solvent. For isoquercitrin (Y_8_), the extraction yield is statistically significant influenced by solvent mixture temperature, percentage of water in the solvent mixture, and the organic solvent. The use of acetone has a positive influence on the recovery of isoquercitrin, while ethanol has a negative influence, both of them being statistically significant. With regard to quercitrin (Y_9_), the most important working parameters were found to be the temperature and, to a lesser extent, the amount of water in the solvent.

For the two phytosterols analyzed, the influence of the extraction conditions is different from those evaluated for the recovery of polyphenols. More precisely, for campesterol (Y_10_) and beta-sitosterol (Y_11_), the highest impact on the extraction yield is attributed to percentage of water in mixture with acetone and ethanol. For both sterols, acetone mixed with a high percentage of water had a positive influence, whereas ethanol displayed a negative influence on the recovery of these two bioactive compounds.

After the analysis of all the evaluated responses and the manner by which each factor influences the extraction yield for the evaluated bioactive compounds, the Modde software generated the optimal extraction conditions for each evaluated bioactive compound, which are given in Table 10. In general, the best working conditions with the highest extraction power for epicatechin, catechin, hyperoside, quercitrin, campesterol, beta-sitosterol are a temperature of 40 °C and a mixture of solvent, acetone and water in equal proportions. 

The phytochemical profile of the analyzed extracts proved that WS can be a valuable source of biologically active compounds for food and/or pharmaceutical industry and warrant the continuation of current research in further evaluating its bioactive potential.

### 3.3. Quantitative Determinations of Total Bioactive Compounds

Numerous studies revealed that phenolic compounds can be found in tree nut species and their health benefits might be attributed to the phenolic profiles and antioxidant activity [49,50]. Most of the phenolic content is found in the tree nut by-products [51], therefore the interest for this research domain. 

Polar solvents are considered the best ones for phenolics extraction, while non-polar solvents (e.g., pentane, hexane, chloroform, diethyl ether) are frequently used for the extraction of less polar constituents, such as tocotrienols and tocopherols, carotenoids and chlorophylls. As expected from previous studies [29,52], the binary-solvent systems extracted more phenolic compounds than the mono-solvent systems. This fact correlates with the differences in the polarity of the extraction mixtures used and solubility of phenolic compounds in them. The mixture of two polar protic solvents, water and ethanol, is less effective than the mixture of a polar protic solvent (water) and a polar, relatively acidic, aprotic solvent (acetone). 

As mentioned before, the UTE method exhibited higher extraction yields than maceration in terms of evaluated bioactive compounds and TAA.

#### 3.3.1. Total Phenolics

A clear difference can be seen between the two richest phenolic compounds extracts based on extraction method: 67.03 ± 9.76 mg GAE/g dw for Ultra-Turrax (run order 5) and 31.27 ± 5.24 mg GAE/g dw for maceration method (run order 11) (Table 3).

We could not find any results for WS in the literature, therefore values for nuts and other by-products were used as comparison. In one study, the TPC in nuts varies from 1.03 to 16.50 mg GAE/g, with pecans, walnuts, and pistachios presenting the highest values [53], while Alasalvar and Bolling (2015) found values at 15.50 to 16.25 mg GAE/g walnut [54]. Another study, performed on walnut seed and by-products, presented TPC mean values of 116.22 ± 3.76 mg GAE/g seed extract (21.43 mg/g walnut seed after taking into account the extraction yields), 94.39 ± 5.63 mg GAE/g leaf extract, and 50.18 ± 2.69 mg GAE/g green husk extract [55]. Akbari et al. (2012) obtained TPC mean values of 52.05 ± 1.27 mg GAE/g, 24.68 ± 0.43 mg GAE/g, and 18.04 ± 0.42 mg GAE/g, for walnut pellicle, hull, and shell, respectively [56], while Shah et al. (2018) acquired quantities from 37.61 to 46.47 mg GAE/g dw walnut leaf extract [57].

Comparing the data from our study with those found in the literature, TPC in WS has equivalent values with those determined for other walnut by-products, such as green husks or leaves. It is evident that WS can be an important source of polyphenolic compounds.

#### 3.3.2. Total Flavonoids

Flavonoids, important polyphenolic compounds in tree nuts, have been associated with several health promoting properties, such as anti-inflammatory, antioxidant, anticancer, antiviral, antibacterial, and hepatoprotective [58]. The highest TFC value was 9.76 ± 0.23 mg QE/g dw walnut septum (run order 14, Table 3), approximately 10 times lower than total extractable phenolics. In one recent study the total flavonoids content of the walnut leaf extract ranged from 5.52 to 28.48 mg QE/g [57], while Mocan et al. (2018), researching *Prunus domestica* leaves, found TFC values between 36.60 ± 2.90 and 60.32 ± 4.12 mg QE/g leaf extract [59]. However, an objective comparison between the results is quite difficult, due to different matrixes and extraction protocols.

#### 3.3.3. Condensed Tannins

Condensed tannins or proanthocyanidins (oligomeric and polymeric forms of flavan-3-ols) are usually quantified using the vanillin assay. The highest CTC value was 237.20 ± 3.22 mg CE/g dw septum (run order 14, Table 3), comparable to those found in pecan nut shell [25,60], but much higher than results obtained in almond kernels [61] or hazelnut and its skin [62]. Knowing that the reactivity of vanillin with catechin is different from that of vanillin with tannins [36], and because the use of catechin as standard in matrices with high content of tannins may under- or overestimate their concentration [60], these values should be viewed with some cautiousness. Despite a potential overestimation, clearly walnut septum is a valuable natural source of condensed tannins. 

Proanthocyanidins are extensively metabolized by gut microbiota to valerolactone intermediates and hydroxybenzoic acids, an important aspect of their bioavailability [63]. A major challenge that influences the bioavailability of these health promoting compounds is their bioaccessibility, the amount which is released from the food matrix in the lumen of the GI tract, and as a result available for absorption [63].

### 3.4. Identification and Quantification of Individual Polyphenols

From the 18 phenolic compounds analyzed by the validated LC-MS/MS method, gentisic acid, *p*-coumaric acid, ferulic acid, hyperosid, isoquercitrin, quercitrin, and quercetin were identified in the WS extracts. Only hyperoside, isoquercitrin, and quercitrin, found in high amounts, were quantified.

All the six polyphenols (epicatechin, catechin, syringic acid, gallic acid, protocatechuic acid, and vanillic acid) analyzed by the other LC-MS method were identified and quantified in WS extracts. This method showed a good linearity (R^2^ > 0.9922) and accuracy (<15%) over the calibration range (Table 2).

After samples hydrolysis, the amount of epicatechin, syringic acid, gallic acid, protocatechuic acid, and vanillic acid increased, while catechin decreased (see Table 11). This registered increase is most probably due to their release from the septum matrix. The particular case of epicatechin increase in parallel with catechin decrease could be attributed to heat-related epimerization of one compound into its optic isomer during the hydrolysis process [26,64].

This is the first study that identifies and quantifies the polyphenolic compounds present in WS.

### 3.5. Identification and Quantification of Phytosterols

The content of phytosterols in the analyzed WS extracts is presented in Table 8. The richest extracts in beta-sitosterol (31.02 mg/g dw septum) and campesterol (0.292 mg/g dw septum) were obtained using 50% aqueous of acetone (run order 7, Table 3). Martinez et al. (2010) identified β-sitosterol at 0.772 to 2.52 mg/g walnut oil and campesterol at 0.044 to 0.121 mg/g walnut oil [65].

### 3.6. Antioxidant Activity

The antioxidant activity of tree nuts or some of their by-products has been previously reported [66,67,68], but there are no references in the literature about WS antioxidant activity. 

Knowing that there are limits in the bioavailability of polyphenols, caused by the extensive catabolism and phase 2 metabolism, it is questionable that polyphenols are responsible for a direct in vivo antioxidant function. However, polyphenols might function through upregulation of antioxidant activity [49]. Ellagitannins, important polyphenols in walnuts with known antioxidant and anti-inflammatory bioactivity, are hydrolyzed to ellagic acid and then converted to urolithins by gut microflora, having a potential role against initiation and progression of several illnesses, including cancer, neurodegenerative, and cardiovascular diseases [27].

#### 3.6.1. ABTS Radical Cation Scavenging Activity Assay

The antioxidant activity against the stable synthetic ABTS radical cation of different WS extracts is summarized in Table 3 and depicted in Figure 2. This assay is based on electron transfer reactions to evaluate radical scavenging activity of various compounds. The highest antioxidant activity was found for the 75% acetone extract (run order 14) at 174.28 ± 8.22 mg TE/g dw septum, followed by the 50% acetone extract (run order 5) at 168.62 ± 9.68 mg TE/g dw septum, both samples obtained by UTE method (Table 3). As mentioned, the antioxidant activity is positively influenced by the fact that these two extracts had the highest content of phenols, flavonoids, and condensed tannins. In other related sources, the ABTS reported scavenging activity was 83.46–93.08% in raw walnuts, 78.3 mg TE/g dw raw pecans, 84.9–93.6% in raw hazelnuts, 309–1375 μmol TE/g hazelnut skin [69], 3.36 mmol TE/g pecan kernel and 8.24 mmol TE/g pecan shell crude extracts [70], 3063–3573 μmol TE/100 g dw natural hazelnut [71]. Nevertheless, because of different ways of expression and/or different preparation method, it is not possible to compare the present results with those from the literature.

#### 3.6.2. DPPH Radical Scavenging Assay

The DPPH (2,2-diphenyl-1-picrylhydrazyl) radical scavenging assay was used to evaluate the ability of septum extracts to scavenge this stable free radical. The change in absorbance at 517 nm is employed as a measure of the scavenging effect of a particular extract for DPPH radicals. The absorbance will decrease faster if the antioxidant activity of the extract (in terms of hydrogen atom-donating capacity) is more potent. Antioxidant molecules can reduce DPPH free radicals and change them to a colorless product resulting in a decreased absorbance [72]. 

In our research, the in vitro DPPH radical scavenging activity was 255.89 mg TE/g of septum extract obtained by Ultra-Turrax with an equal acetone/water volume solution. In other studies on related matrices, the values for the DPPH radical scavenging activity were 14.2 mmol TE/100 g fw (fresh weight) natural walnuts [71], 2.11 mmol TE/g pecan kernel and 4.80 mmol TE/g pecan shell crude extracts [70]. The percentage of DPPH discoloration found by Slatnar et al. (2014) ranged from 60.0 to 96.4% (782.5 to 1682.5 μM trolox/kg) for kernels, 63.0 to 73.2% (312.1 to 810.6 μM trolox/kg) for pellets and 17.7 to 29.9% (870.0 to 1430.2 μM trolox/g) for oil [73]. There are no available data regarding the DPPH assay for WS and a comparison with the results of other researchers, because of different type of samples and measurement units, is not possible.

#### 3.6.3. FRAP (Ferric-Reducing Antioxidant Power) Assay

Reducing power can function as a significant sign of the antioxidant activity and is usually evaluated based on measurement of the conversion of Fe^3+^ to Fe^2+^ in the presence of antioxidants. In our study, the reducing power for the richest polyphenolic septum extract achieved using UTE method and water/acetone (1:1) at 30 °C, was 400.97 mg TE/g septum extract.

As determined by the FRAP assay, the total antioxidant activity of walnut (*Juglans regia*), attributed primarily to their high phenolic content [74], ranked second only to rose hips (*Rosa canina*) among various fruits and foodstuffs [63]. FRAP activity data for related matrices were 418.92 μM Fe^2+^/g to 1067.94 μM Fe^2+^/g fw walnut leaves [57], 95.4 μmol TAE/g to 181.2 μmol TAE/g dw walnut kernel [75], or 454 μmol Fe^2+^/g walnut [76]. As for the other assays, no information was found regarding septum FRAP activity, therefore an objective comparison between the results is unlikely. 

Our results on the topic of the antioxidant action of walnut septum are in agreement with previous reports showing a direct relationship between TPC and antioxidant activity [57,77]. Phenolic compounds can act as free radicals scavengers, hydrogen donors, reducing agents, singlet oxygen quenchers, and metal chelators [55] and are mainly responsible for the walnut antioxidant activity [78]. There are differences in the antioxidant activity between the phenolic compounds most likely due to the number of hydroxyls present in the aromatic ring. Zhang et al. (2009) found that compounds with five hydroxyl groups, such as catechin and epicatechin, were the most active free radical scavengers [79]. These two flavonoids may also present cardiovascular benefits [80], improve blood pressure [81], and positively affect total and low-density lipoprotein cholesterol [82]. Gallic acid, with three hydroxyls showed higher antioxidant activity than protocatechuic acid, with two hydroxyls, or syringic and vanillic acids, with only one hydroxyl [79]. Hyperoside, quercitrin, and isoquercitrin are glycosides formed from the flavonoid quercetin and different types of carbohydrates. Like quercetin, they exhibit antioxidant activity, acting as scavengers of free radicals, but because of the sugar portion of the molecule they are more soluble in water than quercetin, provide superior absorption, and are more bioavailable to the body [83]. In our study, the highest bioactive compounds values recovered from WS extracts via the experimental design correspond to the samples with the highest antioxidant activity, a positive correlation in line with the aforementioned literature findings.

Considering that walnuts possess the highest antioxidant activity in all assays (ABTS, DPPH, FRAP) among nuts [54] and assessing the biological assays of the present study we conclude that WS could be a useful functional ingredient in food technology and pharmaceutical industry.

### 3.7. Tyrosinase Inhibitory Activity

Tyrosinase, a copper-containing enzyme, responsible for the oxidation of tyrosine to l-DOPA and the hydroxylation of l-tyrosine, is involved in several cellular processes, such as biosynthesis of melanin, insect molting, or browning of damaged fruits and vegetables. In humans, melanin regulates skin color and plays a protective role by absorbing ultraviolet sunlight and removing reactive oxygen species from the skin [84]. However, overproduction of melanin in the skin may results in hyperpigmentation or hypermelanosis, characterized by melasma and age spots. Also, over accumulation of melanin in the brain, via oxidation of dopamine, is implicated in the pathogenesis of Parkinson’s disease and related neurodegenerative disorders [85]. Thus, tyrosinase inhibition may not only alleviate skin hyperpigmentation and browning progression in food, but also inhibit wrinkle formation, improve neurodegeneration associated with Parkinson’s disease, and slow down aging [86].

Several recent studies [87,88] aimed to find natural sources of tyrosinase inhibitors in order to replace the synthetic ones, but to the best of our knowledge there is no previous study of tyrosinase inhibitory activity of WS. In the present study, the tyrosinase inhibitory activity of 50% aqueous acetone lyophilized septum extract was 129.98 ± 3.03 mg KAE/g. In other plant matrices, the tyrosinase inhibitory activity was 30.5 ± 1.7 mg KAE/g extract of *Pseudosempervivum* plant [89], 16.81 ± 0.58 mg KAE/g extract of *Lycium* leaves [34], or 31.46 ± 0.19 mg KAE/g extract of *Lycium* berries [90]. Based on these results, we conclude that walnut septum may be a powerful alternative source for natural tyrosinase inhibitors very convenient for the food, pharmaceutical, or cosmetic industry. It can be used to obtain different formulations for preventing the aforementioned disorders.

## 4. Conclusions

This study aimed to characterize septum extracts of walnut (*Juglans regia*) and to describe the optimum experimental conditions for maximizing the extraction efficiency of the bioactive compounds found in this less-studied by-product of this species, in the light of its traditional uses as a remedy for colds and coughs. Specifically, we focused to obtain walnut septum extracts with high content in bioactive compounds (phenols, flavonoids, condensed tannins), having antioxidant and enzyme inhibitory activity, based on an experimental design, and to characterize the phytochemical profile of the extracts using HPLC-MS/MS.

In order to determine the optimal extraction conditions of the main phenolics and phytosterols, several parameters, such as extraction method, solvent, temperature, water percentage, were combined, and they were coupled with statistical tools and chemical analysis (LC-MS/MS). The content in phenolic compounds, tannins, and phytosterols was correlated with the evaluated antioxidant and tyrosinase inhibitory activities. The antioxidant activity of the extracts was assessed using several methods (ABTS, DPPH, and FRAP), and the results showed good antiradical effects. Regarding the tyrosinase inhibitory activity, walnut septum extract showed very good results, therefore, this by-product can be further used in cosmetic products to treat skin hyperpigmentation as well as to inhibit wrinkle formation.

The aforementioned results confirm the use of walnut septum as a health-promoting agent. Further research is needed in order to deeply comprehend the bioavailability of the bioactive molecules and the involved metabolic pathways.

## Figures and Tables

**Table 1:** Independent and dependent variables of experimental design evaluated for walnut septum extracts.

Variables	Level		
	−1	0	1
Independent variables (factors)			
Extraction method (X_1_)	Ultra-turrax		Maceration
Temperature (°C) (X_2_)	20	30	40
Solvent (X_3_)	Acetone		Ethanol
Water in solvent (%, *v*/*v*) (X_4_)	5	25	50
Dependent variables (responses)			
Total phenolic content (TPC, mg GAE/g dw ^1^) (Y_1_)			
Total flavonoid content (TFC, mg QE/g dw ^2^) (Y_2_)			
Condensed tannin content (CTC, mg CE/g dw ^3^) (Y_3_)			
Total antioxidant activity (TAA, mg TE/g dw ^4^) (Y_4_)			

^1^—mg GAE/g dw = gallic acid equivalents per dry weight of walnut septum; ^2^—mg QE/g dw = quercetin equivalents per dry weight of walnut septum; ^3^—mg CE/g dw = catechin equivalents per dry weight of walnut septum; ^4^—mg TE/g dw = trolox equivalents per dry weight of walnut septum.

**Table 2:** Detection and quantification of certain polyphenols by the new LC-MS method developed in view of their analysis in walnut septum extracts.

Polyphenol	Monitored Ion (*m*/*z*)	Retention Time (min)	Calibration Range (*n* = 8) (µg/mL)	Coefficient of Linearity (R^2^)	Accuracy (Bias, %)
Epicatechin	289	9.0	0.3–21.5	0.9922	90.7–112.1
Catechin	289	6.0	0.3–21.5	0.9974	94.3–108.9
Gallic acid	169	1.5	0.3–22.2	0.9987	96.4–108.6
Syringic acid	197	8.4	0.3–21.0	0.9997	90.5–105.5
Protocatechuic acid	153	2.8	0.3–23.9	0.9977	87.0–112.2
Vanillic acid	167	6.7	0.3–21.1	0.9993	95.6–105.6

**Table 3:** Matrix of experimental design and experimental results for total phenolic content (TPC), total flavonoid content (TFC), condensed tannin content (CTC), and total antioxidant activity (TAA) of walnut septum extracts based on a factorial design.

Sample Code	Run Order	Factorial Design with Coded Values				Determination (Experimental Results)			
		X_1_	X_2_	X_3_	X_4_	Y_1_ (TPC)	Y_2_ (TFC)	Y_3_ (CTC)	Y_4_ (TAA)
N1	9	Ultra-turrax	40	Acetone	5	32.60 ± 1.24	3.91 ± 0.18	126.70 ± 0.74	89.69 ± 0.48
N2	13	Ultra-turrax	20	Acetone	5	14.01 ± 1.53	1.85 ± 0.07	63.97 ± 0.63	39.55 ± 1.45
N3	17	Ultra-turrax	30	Acetone	25	50.51 ± 3.55	7.61 ± 0.64	181.74 ± 1.11	146.51 ± 2.40
N4	14	Ultra-turrax	30	Acetone	25	59.52 ± 10.99	9.76 ± 0.23	237.20 ± 3.22	174.28 ± 8.22
N5	10	Ultra-turrax	30	Acetone	25	61.75 ± 5.30	9.12 ± 1.11	227.71 ± 0.71	163.46 ± 4.42
N6	20	Ultra-turrax	30	Acetone	25	28.62 ± 1.20	4.04 ± 0.13	56.60 ± 0.56	55.51 ± 11.84
N7	18	Ultra-turrax	20	Acetone	50	34.80 ± 5.32	5.81 ± 0.07	74.04 ± 0.81	101.28 ± 2.58
N8	5	Ultra-turrax	40	Acetone	50	67.03 ± 9.76	8.99 ± 0.09	235.77 ± 7.47	168.62 ± 9.68
N9	6	Ultra-turrax	20	Ethanol	5	18.10 ± 1.46	4.08 ± 1.71	85.81 ± 0.16	61.14 ± 2.74
N10	19	Ultra-turrax	27	Ethanol	5	34.65 ± 0.96	4.79 ± 0.48	156.77 ± 0.14	102.77 ± 4.31
N11	15	Ultra-turrax	40	Ethanol	5	48.37 ± 3.90	7.05 ± 1.96	184.07 ± 1.36	122.35 ± 2.18
N12	4	Ultra-turrax	40	Ethanol	50	22.80 ± 1.89	2.40 ± 0.13	37.70 ± 0.03	41.53 ± 5.27
N13	16	Ultra-turrax	20	Ethanol	50	45.03 ± 2.64	6.51 ± 0.39	131.92 ± 0.22	120.18 ± 3.01
N14	22	Maceration	20	Acetone	5	13.29 ± 0.48	1.20 ± 0.04	2.98 ± 0.13	11.21 ± 0.61
N15	23	Maceration	40	Acetone	5	6.64 ± 4.26	0.82 ± 0.05	17.89 ± 0.11	17.67 ± 0.38
N16	8	Maceration	30	Acetone	25	24.37 ± 1.64	5.53 ± 0.06	16.60 ± 0.06	68.47 ± 1.66
N17	11	Maceration	40	Acetone	50	31.27 ± 5.24	7.11 ± 0.19	15.05 ± 0.52	77.68 ± 7.89
N18	3	Maceration	20	Acetone	50	13.97 ± 2.53	1.84 ± 0.04	55.14 ± 0.16	41.23 ± 0.14
N19	21	Maceration	40	Ethanol	5	17.27 ± 2.43	2.04 ± 0.42	1.14 ± 0.04	20.66 ± 3.47
N20	7	Maceration	20	Ethanol	5	25.04 ± 2.50	2.86 ± 0.24	86.90 ± 0.50	58.66 ± 1.52
N21	1	Maceration	40	Ethanol	33	14.30 ± 2.89	2.10 ± 0.06	28.09 ± 0.06	36.22 ± 0.59
N22	12	Maceration	20	Ethanol	50	29.08 ± 5.01	6.13 ± 0.15	18.18 ± 0.03	82.36 ± 1.49
N23	2	Maceration	40	Ethanol	50	16.63 ± 5.59	4.78 ± 2.75	2.26 ± 0.09	23.02 ± 3.38

X_1_, extraction method; X_2_, temperature (°C); X_3_, solvent; X_4_, water in solvent (%, *v*/*v*). Y_1_, TPC—total phenolic content expressed as mg GAE/g dw = gallic acid equivalents per dry weight of walnut septum; Y_2_, TFC—total flavonoid content expressed as mg QE/g dw = quercetin equivalents per dry weight of walnut septum; Y_3_, CTC—condensed tannin content expressed as mg CE/g dw = catechin equivalents per dry weight of walnut septum; Y_4_, TAA—total antioxidant activity expressed as mg TE/g dw = trolox equivalents per dry weight of walnut septum. Data are shown as mean ± SD (standard deviation).

**Table 4:** Optimization of extraction parameter for fitted factorial model by analysis of variance (ANOVA).

Quantifiable Responses	Reproducibility	Source	Degrees of Freedom	Sum of Squares	Mean Square	*F* Value	*p* Value
Total phenolic content (Y_1_) (R^2^ = 0.75, Q^2^ = 0.52)	0.86	Regression	8	4838.0	604.7	4.90	0.006
		Lack of fit	10	1481.1	148.1	3.60	0.159
		Pure error	3	123.2	41.05		
Total flavonoid content (Y_2_) (R^2^ = 0.61, Q^2^ = 0.37)	0.88	Regression	7	97.52	13.93	3.26	0.028
		Lack of fit	11	57.09	5.19	5.79	0.087
		Pure error	3	2.68	0.89		
Condensed tannin content (Y_3_) (R^2^ = 0.80, Q^2^ = 0.63)	0.82	Regression	7	112,775	16,110.8	8.29	0.001
		Lack of fit	11	23,769.4	2160.8	1.88	0.329
		Pure error	3	3435.2	1145.8		
Total antioxidant activity (Y_4_) (R^2^ = 0.75, Q^2^ = 0.59)	0.92	Regression	7	41,969.5	5995.6	6.21	0.002
		Lack of fit	11	12,939.3	1176.3	6.18	0.080
		Pure error	3	570.4	190.1		

R^2^, coefficient of determination; *F*-value, Fischer’s ratio; *p*-value, probability; Q^2^, goodness of prediction.

**Table 5:** Regression equation coefficients.

Effect	Responses			
	Y_1_ (Total Phenolic Content)	Y_2_ (Total Flavonoid Content)	Y_3_ (Condensed Tannin Content)	Y_4_ (Total Antioxidant Activity)
Constant	38.859	4.844	92.917	80.521
X_1_ (M)	−10.595	−1.281	−63.611	−33.412
X_1_ (UTE)	10.595	1.281	63.611	33.412
X_2_ (Temperature)	1.551	0.2914	1.763	1.892
X_3_ (Acetone)	1.737	0.4991	14.528	11.771
X_3_ (Ethanol)	−1.737	−0.4991	−14.528	−11.771
X_4_ (Water %)	5.534	1.196	5.550	14.822
X_4_ × X_4_	−8.261	-	-	-
X_1_ (M) × X_2_	-	-	−12.390	-
X_1_ (UTE) × X_2_	-	-	12.390	-
X_2_ × X_3_ (Acetone)	4.818	0.7329	-	13.769
X_2_ × X_3_ (Ethanol)	−4.819	−0.7329	-	13.769
X_1_ (M) × X_3_ (Acetone)	−3.291	−0.6606	−20.223	−13.364
X_1_ (M) × X_3_ (Ethanol)	3.291	0.6606	20.223	13.364
X_1_ (UTE) × X_3_ (Acetone)	3.291	0.6606	20.223	13.364
X_1_ (UTE) × X_3_ (Ethanol)	−3.291	−0.6606	−20.223	−13.364
X_3_ (Acetone) × X_4_	5.023	0.8522	26.089	15.728
X_3_ (Ethanol) × X_4_	−5.023	−0.8522	−26.089	−15.728

M—maceration; UTE—ultra-turrax extraction. For data in bold, *p*-value was <0.005, therefore statistically significant.

**Table 6:** Independent and dependent variable of experimental design evaluated for bioactive compounds from walnut septum extracts.

Variables	Level		
	−1	0	1
Independent variables (factors)			
Temperature (°C) (X_1_)	20	30	40
Solvent (%, *v*/*v*) (X_2_)	Acetone		Ethanol
Water in solvent (%, *v*/*v*) (X_3_)	5	25	50
Dependent variables (responses)			
Epicatechin (μg/g dw) (Y_1_)			
Catechin (μg/g dw) (Y_2_)			
Syringic acid (μg/g dw) (Y_3_)			
Syringic acid (μg/g dw) (Y_3_)			
Gallic acid (μg/g dw) (Y_4_)			
Protocatechuic acid (μg/g dw) (Y_5_)			
Vanillic acid (μg/g dw) (Y_6_)			
Hyperoside (μg/g dw) (Y_7_)			
Isoquercitrin (μg/g dw) (Y_8_)			
Quercitrin (μg/g dw) (Y_9_)			
Campesterol (μg/g dw) (Y_10_)			
Beta-sitosterol (μg/g dw) (Y_11_)			

All units are expressed as μg identified compound per gram of dry weight walnut extract.

**Table 7:** Matrix of experimental design for bioactive compounds recovery from walnut septum extracts.

Sample Code	Run Order	Factorial Design with Coded Values			Determination (Experimental Results)										
		X_1_	X_2_	X_3_	Y_1_	Y_2_	Y_3_	Y_4_	Y_5_	Y_6_	Y_7_	Y_8_	Y_9_	Y_10_	Y_11_
N1	9	40	Acetone	5	6.091	288.29	2.392	29.074	3.441	2.229	32.726	71.290	583.86	106.22	9932.57
N2	13	20	Acetone	5	2.703	138.59	1.021	13.655	2.117	1.680	13.110	24.039	216.02	42.702	9080.36
N3	17	30	Acetone	25	10.493	447.67	4.215	55.240	13.388	6.219	46.858	99.334	980.69	131.36	26,461.16
N4	14	30	Acetone	25	11.463	468.62	5.010	61.002	11.575	8.881	43.083	109.42	894.89	140.26	19,546.42
N5	10	30	Acetone	25	8.540	408.01	5.154	48.422	12.845	7.173	40.641	94.494	852.79	162.52	22,146.69
N6	20	30	Acetone	25	10.093	396.95	7.111	148.16	28.108	8.905	35.702	68.449	694.32	36.067	5929.59
N7	18	20	Acetone	50	5.136	250.65	3.221	31.031	8.427	3.537	24.933	54.283	495.81	ND	1338.98
N8	5	40	Acetone	50	12.540	597.65	5.202	79.584	9.943	5.577	67.329	103.60	1073.04	292.07	31,018.16
N9	6	20	Ethanol	5	3.533	152.11	1.560	16.679	2.498	1.723	19.014	37.562	326.99	114.36	15,243.10
N10	19	26	Ethanol	5	6.861	273.05	2.381	27.903	4.400	3.022	36.926	75.113	629.22	162.84	22,277.34
N11	15	40	Ethanol	5	8.556	329.04	2.853	31.983	9.645	5.728	33.095	70.960	695.23	8.988	1175.42
N12	4	40	Ethanol	50	9.800	596.98	14.711	130.95	138.58	40.277	32.288	77.528	867.83	104.04	21,736.13
N13	16	20	Ethanol	50	3.238	79.930	6.860	63.446	86.115	11.476	28.768	49.137	449.96	171.06	28,934.75

X_1_, temperature (°C); X_2_, solvent; X_3_, water in solvent (%, *v*/*v*). Y_1_—Epicatechin; Y_2_—Catechin; Y_3_—Syringic acid; Y_4_—Gallic acid; Y_5_—Protocatechuic acid; Y_6_—Vanillic acid; Y_7_—Hyperoside; Y_8_—Isoquercitrin; Y_9_—Quercitrin; Y_10_—Campesterol; Y_11_—Beta-sitosterol. All responses are expressed as μg bioactive compound per gram of dry weight walnut septum. ND—not determined.

**Table 8:** Optimization of extraction parameter for fitted factorial model by analysis of variance for bioactive compounds in walnut septum extracts (ANOVA).

Quantifiable Responses	Reproducibility	Source	Degrees of Freedom	Sum of Squares	Mean Square	*F* Value	*p* Value
Epicatechin (Y_1_) (R^2^ = 0.91, Q^2^ = 0.55)	0.86	Regression	5	1.18 × 10^−4^	2.36 × 10^−5^	16.040	0.001
		Lack of fit	4	5.88 × 10^−6^	1.47 × 10^−6^	0.9956	0.523
		Pure error	3	4.43 × 10^−6^	1.48 × 10^−6^		
Catechin (Y_2_) (R^2^ = 0.94, Q^2^ = 0.65)	0.95	Regression	5	3.09 × 10^−1^	6.19 × 10^−2^	24.345	0.001
		Lack of fit	4	1.44 × 10^−2^	3.60 × 10^−3^	3.1984	0.183
		Pure error	3	3.38 × 10^−3^	1.13 × 10^−3^		
Syringic acid (Y_3_) (R^2^ = 0.79, Q^2^ = 0.44)	0.87	Regression	4	1.19 × 10^−4^	2.99 × 10^−5^	7.5251	0.008
		Lack of fit	5	2.72 × 10^−5^	5.44 × 10^−6^	3.5928	0.161
		Pure error	3	4.54 × 10^−6^	1.51 × 10^−6^		
Gallic acid (Y_4_) (R^2^ = 0.97, Q^2^ = 0.67)	0.96	Regression	6	1.15 × 10^−2^	1.92 × 10^−3^	37.712	0.001
		Lack of fit	3	1.76 × 10^−4^	5.85 × 10^−5^	1.4762	0.428
		Pure error	2	7.93 × 10^−5^	3.97 × 10^−5^		
Protocatechuic acid (Y_5_) (R^2^ = 0.93, Q^2^ = 0.68)	0.96	Regression	4	1.84 × 10^−2^	4.60 × 10^−3^	27.368	0.001
		Lack of fit	5	1.16 × 10^−3^	2.31 × 10^−4^	3.6946	0.156
		Pure error	3	1.88 × 10^−4^	6.26 × 10^−5^		
Vanillic acid (Y_6_) (R^2^ = 0.84, Q^2^ = 0.19)	0.81	Regression	5	8.37 × 10^−5^	1.67 × 10^−5^	5.4394	0.043
		Lack of fit	3	1.18 × 10^−5^	3.92 × 10^−6^	2.1522	0.333
		Pure error	2	3.64 × 10^−6^	1.82 × 10^−6^		
Hyperoside (Y_7_) (R^2^ = 0.88, Q^2^ = 0.34)	0.87	Regression	6	1.93 × 10^−3^	3.21 × 10^−4^	7.6743	0.013
		Lack of fit	3	1.86 × 10^−4^	6.19 × 10^−5^	2.8308	0.208
		Pure error	3	6.55 × 10^−5^	2.18 × 10^−5^		
Isoquercitrin (Y_8_) (R^2^ = 0.98, Q^2^ = 0.76)	0.92	Regression	6	7.86 × 10^−3^	1.31 × 10^−3^	41.990	0.001
		Lack of fit	3	4.00 × 10^−5^	1.33 × 10^−5^	0.2295	0.870
		Pure error	2	1.16 × 10^−4^	5.80 × 10^−5^		
Quercitrin (Y_9_) (R^2^ = 0.92, Q^2^ = 0.63)	0.78	Regression	5	7.32 × 10^−1^	1.46 × 10^−1^	16.425	0.001
		Lack of fit	4	1.92 × 10^−2^	4.80 × 10^−3^	0.3334	0.842
		Pure error	3	4.32 × 10^−2^	1.44 × 10^−2^		
Campesterol (Y_10_) (R^2^ = 0.91, Q^2^ = 0.34)	0.95	Regression	6	6.34 × 10^−2^	1.06 × 10^−2^	8.6364	0.016
		Lack of fit	3	5.61 × 10^−3^	1.87 × 10^−3^	7.2591	0.123
		Pure error	2	5.15 × 10^−4^	2.57 × 10^−4^		
Beta-sitosterol (Y_11_) (R^2^ = 0.87, Q^2^ = 0.25)	0.88	Regression	6	9.76 × 10^2^	1.63 × 10^2^	5.6232	0.039
		Lack of fit	3	1.20 × 10^2^	40.1	3.2875	0.242
		Pure error	2	24.4	12.2		

R^2^, coefficient of determination; *F*-value, Fischer’s ratio; *p*-value, probability. Q^2^, goodness of prediction.

**Table 9:** Regression equation coefficients for bioactive compounds determined in walnut septum extracts.

Effect	Response										
	Y_1_	Y_2_	Y_3_	Y_4_	Y_5_	Y_6_	Y_7_	Y_8_	Y_9_	Y_10_	Y_11_
	Epicatechin	Catechin	Syringic Acid	Gallic Acid	Protocatechuic Acid	Vanillic Acid	Hyper oside	Iso quercitrin	Quercitrin	Campesterol	Beta-sitosterol
Constant	**0.00936**	**0.0244**	**0.00489**	**0.0589**	**0.0279**	**0.007207**	**0.03906**	**0.0947**	**0.815**	**0.141**	**22.6**
X_1_ (Temperature)	**0.00257**	**0.125**	**0.00127**	**0.0157**	0.00618	0.00154	**0.00968**	**0.02044**	**0.198**	0.0277	2.13
X_2_ (Acetone)	0.000325	0.0153	−0.00115	**−0.008201**	**−0.0223**	**−0.00173**	0.00214	0.00286	0.01066	−0.000295	−1.91
X_2_ (Ethanol)	−0.000325	−0.0153	0.00115	**0.008201**	**0.0223**	**0.00173**	−0.00214	−0.00286	−0.01066	0.000295	1.91
X_3_ (Water %)	**0.00117**	**0.0612**	**0.00197**	**0.0223**	**0.01902**	**0.00245**	**0.00623**	**0.00977**	**0.116**	**0.0295**	**5.31**
X_1_ × X_1_	**−0.00199**	**−0.0679**	-	**−0.01015**	-	−0.00175	−0.00522	**−0.0253**	**−0.159**	−0.0275	−6.206
X_1_ × X_3_	-	**0.05056**	-	**0.00972**	-	-	-	-	-	**0.0285**	3.75
X_1_ × X_2_ (Acetone)	-	-	-	-	-	-	**0.004901**	0.00397	-	**0.0593**	**5.77**
X_1_ × X_2_ (Ethanol)	-	-	-	-	-	-	**−0.004901**	−0.00397	-	**−0.0593**	**−5.77**
X_2_ (Acetone) × X_3_	0.000853	-	−0.00133	**−0.00896**	**−0.0219**	−0.00153	0.00461	**0.00541**	0.0576	-	-
X_2_ (Ethanol) × X_3_	−0.000853	-	0.00133	**0.00896**	**0.0219**	0.00153	−0.00461	**−0.00541**	−0.576	-	-

For data in bold, *p*-value was <0.005, therefore statistically significant.

**Table 10:** Optimum experimental conditions for improved recovery of bioactive compounds from walnut septum extracts obtained by ultra-turrax extraction.

Evaluated	TPC ^1^	TFC ^2^	CTC ^3^	TAA ^4^	Epi- catechin	Catechin	Syringic Acid	Gallic Acid	Proto- catechuic Acid	Vanillic Acid	Hyper- oside	Iso- quercitrin	Quercitrin	Campesterol	Beta-sitosterol
**Temperature**	40 °C	30 °C	30 °C	30 °C	40 °C	40 °C	40 °C	30 °C	40 °C	40 °C	40 °C	30 °C	40 °C	40 °C	40 °C
**Solvent**	Acetone	Acetone	Acetone	Acetone	Acetone	Acetone	Ethanol	Acetone	Ethanol	Ethanol	Acetone	Acetone	Acetone	Acetone	Acetone
**Water %**	50%	25%	25%	25%	50%	50%	50%	25%	50%	50%	50%	25%	50%	50%	50%
**Determined**	67.03 ± 9.76	9.76 ± 0.23	237.20 ± 3.22	174.28 ± 8.22	12.450	597.647	14.711	148.164	138.58	40.277	67.329	109.42	1073.04	292.07	31018.16

^1^ TPC—total phenolic content expressed as mg GAE/g dw = gallic acid equivalents per dry weight of walnut septum; ^2^ TFC—total flavonoid content expressed as mg QE/g dw = quercetin equivalents per dry weight of walnut septum; ^3^ CTC—condensed tannin content expressed as mg CE/g dw = catechin equivalents per dry weight of walnut septum; ^4^ TAA—total antioxidant activity expressed as mg TE/g dw = trolox equivalents per dry weight of walnut septum. Data are shown as mean ± SD (standard deviation). All determined amount of bioactive compounds are expressed as μg bioactive compound per gram of dry weight walnut septum.

**Table 11:** Quantitative evaluation of the recovery of main bioactive compounds in non-hydrolyzed and hydrolyzed samples of walnut septum extracts.

Sample Code/Bioactive Compound	Non-Hydrolyzed Sample						Hydrolyzed Samples					
	Epicatechin	Catechin	Syringic Acid	Gallic Acid	Protocatechuic Acid	Vanillic Acid	Epicatechin	Catechin	Syringic Acid	Gallic Acid	Protocatechuic Acid	Vanillic Acid
N1	0.006	0.288	0.002	0.029	0.003	0.002	0.249	0.282	0.047	1.918	0.065	0.036
N2	0.003	0.139	0.001	0.014	0.002	0.002	0.097	0.089	0.031	1.084	0.037	0.027
N3	0.010	0.448	0.004	0.055	0.013	0.006	0.356	0.377	0.088	3.537	0.132	0.097
N4	0.011	0.469	0.005	0.061	0.012	0.009	0.134	0.083	0.096	4.194	0.100	0.092
N5	0.009	0.408	0.005	0.048	0.013	0.007	0.316	0.286	0.091	3.543	0.145	0.103
N6	0.010	0.397	0.007	0.148	0.028	0.009	0.247	0.244	0.099	3.814	0.168	0.128
N7	0.005	0.251	0.003	0.031	0.008	0.004	0.266	0.311	0.044	1.943	0.074	0.053
N8	0.013	0.598	0.005	0.080	0.010	0.006	0.544	0.555	0.108	4.436	0.140	0.071
N9	0.004	0.152	0.002	0.017	0.002	0.002	0.149	0.146	0.031	1.447	0.040	0.027
N10	0.007	0.273	0.002	0.028	0.004	0.003	0.227	0.215	0.063	2.484	0.090	0.056
N11	0.009	0.329	0.003	0.032	0.010	0.006	0.254	0.273	0.064	2.487	0.101	0.069
N12	0.010	0.597	0.015	0.131	0.139	0.040	0.141	0.156	0.110	2.738	0.277	0.130
N13	0.003	0.080	0.007	0.063	0.086	0.011	0.000	0.037	0.046	1.622	0.223	0.076

All determined amount of bioactive compounds are expressed as mg bioactive compound per gram of dry weight walnut septum.

## References

[1] Soccol C.R., da Costa E.S.F., Letti L.A.J., Karp S.G., Woiciechowski A.L., de Souza Vandenberghe L.P. (2017). Recent developments and innovations in solid state fermentation. Biotechnol. Res. Innov..

[2] Zhang S., Wang S., Huang J., Lai X., Du Y., Liu X., Li B., Feng R., Yang G. (2016). High-specificity quantification method for almond-by-products, based on differential proteomic analysis. Food Chem..

[3] Kempf K., Martin S., Döhring C., Dugi K., von Wolmar C.W., Haastert B., Schneider M. (2013). The epidemiological Boehringer Ingelheim Employee study-part I: Impact of overweight and obesity on cardiometabolic risk. J. Obes..

[4] Murakami K., Livingstone M.B.E. (2015). Eating Frequency Is Positively Associated with Overweight and Central Obesity in US Adults. J. Nutr..

[5] Franceschi C., Campisi J. (2014). Chronic inflammation (Inflammaging) and its potential contribution to age-associated diseases. J. Gerontol. A Biol. Sci. Med. Sci..

[6] Leon-Cabrera S., Solís-Lozano L., Suárez-Álvarez K., González-Chávez A., Béjar Y.L., Robles-Díaz G., Escobedo G. (2013). Hyperleptinemia is associated with parameters of low-grade systemic inflammation and metabolic dysfunction in obese human beings. Front. Integr. Neurosci..

[7] Garg S.K., Maurer H., Reed K., Selagamsetty R. (2014). Diabetes and cancer: Two diseases with obesity as a common risk factor. Diabetes Obes. Metab..

[8] Bastien M., Poirier P., Lemieux I., Després J.P. (2014). Overview of epidemiology and contribution of obesity to cardiovascular disease. Prog. Cardiovasc. Dis..

[9] Divella R., De Luca R., Abbate I., Naglieri E., Daniele A. (2016). Obesity and cancer: The role of adipose tissue and adipo-cytokines-induced chronic inflammation. J. Cancer.

[10] Mauland K.K., Eng Ø., Ytre-Hauge S., Tangen I.L., Berg A., Salvesen H.B., Salvesen Ø.O., Krakstad C., Trovik J., Hoivik E.A. (2017). High visceral fat percentage is associated with poor outcome in endometrial cancer. Oncotarget.

[11] Gui Y., Pan Q., Chen X., Xu S., Luo X., Chen L. (2017). The association between obesity related adipokines and risk of breast cancer: A meta-analysis. Oncotarget.

[12] Cani P.D., Jordan B.F. (2018). Gut microbiota-mediated inflammation in obesity: A link with gastrointestinal cancer. Nat. Rev. Gastroenterol. Hepatol..

[13] Eibl G., Cruz-Monserrate Z., Korc M., Petrov M.S., Goodarzi M.O., Fisher W.E., Habtezion A., Lugea A., Pandol S.J., Hart P.A. (2018). Diabetes Mellitus and Obesity as Risk Factors for Pancreatic Cancer. J. Acad. Nutr. Diet..

[14] Ferro M., Terracciano D., Buonerba C., Lucarelli G., Bottero D., Perdonà S., Autorino R., Serino A., Cantiello F., Damiano R. (2017). The emerging role of obesity, diet and lipid metabolism in prostate cancer. Future Oncol..

[15] Petrick J., Freedman N., Demuth J., Yang B., Van Den Eeden S., Engel L., McGlynn K. (2016). Obesity, diabetes, serum glucose, and risk of primary liver cancer by birth cohort, race/ethnicity, and sex: Multiphasic health checkup study. Cancer Epidemiol..

[16] Zhang J., Chen Q., Li Z.-M., Xu X.-D., Song A.-F., Wang L.-S. (2018). Association of body mass index with mortality and postoperative survival in renal cell cancer patients, a meta-analysis. Oncotarget.

[17] Aravani A., Downing A., Thomas J.D., Lagergren J., Morris E.J.A., Hull M.A. (2018). Obesity surgery and risk of colorectal and other obesity-related cancers: An English population-based cohort study. Cancer Epidemiol..

[18] Grosso G., Yang J., Marventano S., Micek A., Galvano F., Kales S. (2015). Nut consumption on all-cause, cardiovascular, and cancer mortality risk: A systematic review and meta-analysis of epidemiologic studies. Am. J. Clin. Nutr..

[19] Aune D., Keum N., Giovannucci E., Fadnes L., Boffetta P., Greenwood D., Tonstad S., Vatten L., Riboli E., Norat T. (2016). Nut consumption and risk of cardiovascular disease, total cancer, all-cause and cause-specific mortality: A systematic review and dose-response meta-analysis of prospective studies. BMC Med..

[20] Hever J., Cronise R.J. (2017). Plant-based nutrition for healthcare professionals: Implementing diet as a primary modality in the prevention and treatment of chronic disease. J. Geriatr. Cardiol..

[21] Shahidi F., Ambigaipalan P. (2015). Phenolics and polyphenolics in foods, beverages and spices: Antioxidant activity and health effects-A review. J. Funct. Foods.

[22] Oroian M., Escriche I. (2015). Antioxidants: Characterization, natural sources, extraction and analysis. Food Res. Int..

[23] Bjørklund G., Chirumbolo S. (2017). Role of oxidative stress and antioxidants in daily nutrition and human health. Nutrition.

[24] Smeriglio A., Denaro M., Barreca D., Calderaro A., Bisignano C., Ginestra G., Bellocco E., Trombetta D. (2017). In vitro evaluation of the antioxidant, cytoprotective, and antimicrobial properties of essential oil from *Pistacia vera* L. Variety Bronte Hull. Int. J. Mol. Sci..

[25] do Prado A., da Silva H., da Silveira S., Barreto P., Vieira C., Maraschin M., Ferreira S., Block J. (2014). Effect of the extraction process on the phenolic compounds profile and the antioxidant and antimicrobial activity of extracts of pecan nut [*Carya illinoinensis* (Wangenh) C. Koch] shell. Ind. Crop. Prod..

[26] Rusu M.E., Gheldiu A.-M., Mocan A., Vlase L., Popa D.-S. (2018). Anti-aging potential of tree nuts with a focus on phytochemical composition, molecular mechanisms and thermal stability of major bioactive compounds. Food Funct..

[27] Sánchez-González C., Ciudad C.J., Noé V., Izquierdo-Pulido M. (2017). Health benefits of walnut polyphenols: An exploration beyond their lipid profile. Crit. Rev. Food Sci. Nutr..

[28] Vieira V., Prieto M.A., Barros L., Coutinho J.A.P., Ferreira O., Ferreira I.C.F.R. (2017). Optimization and comparison of maceration and microwave extraction systems for the production of phenolic compounds from *Juglans regia* L. for the valorization of walnut leaves. Ind. Crops Prod..

[29] Fernández-Agulló A., Pereira E., Freire M.S., Valentão P., Andrade P.B., González-Álvarez J., Pereira J.A. (2013). Influence of solvent on the antioxidant and antimicrobial properties of walnut (*Juglans regia* L.) green husk extracts. Ind. Crop. Prod..

[30] Dehghani F., Mashhoody T., Panjehshahin M. (2012). Effect of aqueous extract of walnut septum on blood glucose and pancreatic structure in streptozotocin-induced diabetic mouse. Iran. J. Pharmacol. Ther..

[31] Ramishvili L., Gordeziani M., Tavdishvili E., Bedineishvili N., Dzidziguri D., Kotrikadze N. (2016). The effect of extract of greek walnut (*Juglans regia* L.) septa on some functional characteristics of erythrocytes. Georg. Med. News.

[32] Ravanbakhsh A., Mahdavi M., Jalilzade-Amin G., Javadi S., Maham M., Mohammadnejad D., Rashidi M.R. (2016). Acute and subchronic toxicity study of the median septum of *Juglans regia* in Wistar rats. Adv. Pharm. Bull..

[33] Christopoulos M., Tsantili E. (2012). Storage of fresh walnuts (*Juglans regia* L.)-Low temperature and phenolic compounds. Postharvest Biol. Technol..

[34] Mocan A., Zengin G., Simirgiotis M., Schafberg M., Mollica A., Vodnar D.C., Crişan G., Rohn S. (2017). Functional constituents of wild and cultivated Goji (*L. barbarum* L.) leaves: Phytochemical characterization, biological profile, and computational studies. J. Enzym. Inhib. Med. Chem..

[35] Mocan A., Schafberg M., Crisan G., Rohn S. (2016). Determination of lignans and phenolic components of *Schisandra chinensis* (Turcz.) Baill. using HPLC-ESI-ToF-MS and HPLC-online TEAC: Contribution of individual components to overall antioxidant activity and comparison with traditional antioxidant assays. J. Funct. Foods.

[36] Price M.L., Van Scoyoc S., Butler L.G. (1978). A Critical Evaluation of the Vanillin Reaction as an Assay for Tannin in Sorghum Grain. J. Agric. Food Chem..

[37] Alasalvar C., Karamać M., Amarowicz R., Shahidi F. (2006). Antioxidant and antiradical activities in extracts of hazelnut kernel (*Corylus avellana* L.) and hazelnut green leafy cover. J. Agric. Food Chem..

[38] Meda R.N., Vlase L., Lamien-Meda A., Lamien C.E., Muntean D., Tiperciuc B., Oniga I., Nacoulma O.G. (2011). Identification and quantification of phenolic compounds from *Balanites aegyptiaca* (L.) Del (Balanitaceae) galls and leaves by HPLC-MS. Nat. Prod. Res..

[39] Mocan A., Vlase L., Raita O., Hanganu D., Paltinean R., Dezsi S., Gheldiu A.M., Oprean R., Crisan G. (2015). Comparative studies on antioxidant activity and polyphenolic content of *Lycium barbarum* L. and *Lycium chinense* Mill. leaves. Pak. J. Pharm. Sci..

[40] Pop C.E., Pârvu M., Arsene A.L., Pârvu A.E., Vodnar D.C., Tarcea M., Toiu A.M., Vlase L. (2017). Investigation of antioxidant and antimicrobial potential of some extracts from *Hedera helix* L.. Farmacia.

[41] Babotă M., Mocan A., Vlase L., Crisan O., Ielciu I., Gheldiu A.M., Vodnar D.C., Crişan G., Păltinean R. (2018). Phytochemical analysis, antioxidant and antimicrobial activities of *Helichrysum arenarium* (L.) Moench. and *Antennaria dioica* (L.) Gaertn. flowers. Molecules.

[42] Vlase L., Parvu M., Parvu E.A., Toiu A. (2012). Phytochemical analysis of *Allium fistulosum* L. and *A. ursinum* L.. Dig. J. Nanomater. Biostruct..

[43] Toiu A., Mocan A., Vlase L., Pârvu A.E., Vodnar D.C., Gheldiu A.M., Moldovan C., Oniga I. (2018). Phytochemical composition, antioxidant, antimicrobial and in vivo anti-inflammatory activity of traditionally used Romanian *Ajuga laxmannii* (Murray) Benth. (“Nobleman’s beard”-barba împăratului). Front. Pharmacol..

[44] Shahidi F., Alasalvar C., Liyana-Pathirana C.M. (2007). Antioxidant phytochemicals in hazelnut kernel (*Corylus avellana* L.) and hazelnut byproducts. J. Agric. Food Chem..

[45] Mocan A., Fernandes Â., Barros L., Crişan G., Smiljković M., Soković M., Ferreira I.C.F.R. (2018). Chemical composition and bioactive properties of the wild mushroom: *Polyporus squamosus* (Huds.) Fr: A study with samples from Romania. Food Funct..

[46] Damiano S., Forino M., De A., Vitali L.A., Lupidi G., Taglialatela-Scafati O. (2017). Antioxidant and antibiofilm activities of secondary metabolites from *Ziziphus jujuba* leaves used for infusion preparation. Food Chem..

[47] Masuda T., Fujita N., Odaka Y., Takeda Y., Yonemori S., Nakamoto K., Kuninaga H. (2007). Tyrosinase inhibitory activity of ethanol extracts from medicinal and edible plants cultivated in okinawa and identification of a water-soluble inhibitor from the leaves of *Nandina domestica*. Biosci. Biotechnol. Biochem..

[48] Hilbig J., Alves V.R., Müller C.M.O., Micke G.A., Vitali L., Pedrosa R.C., Block J.M. (2018). Ultrasonic-assisted extraction combined with sample preparation and analysis using LC-ESI-MS/MS allowed the identification of 24 new phenolic compounds in pecan nut shell [*Carya illinoinensis* (Wangenh) C. Koch] extracts. Food Res. Int..

[49] Bolling B.W. (2017). Almond Polyphenols: Methods of Analysis, Contribution to Food Quality, and Health Promotion. Compr. Rev. Food Sci. Food Saf..

[50] Esposito T., Sansone F., Franceschelli S., Del Gaudio P., Picerno P., Aquino R.P., Mencherini T. (2017). Hazelnut (*Corylus avellana* L.) Shells Extract: Phenolic Composition, Antioxidant Effect and Cytotoxic Activity on Human Cancer Cell Lines. Int J. Mol. Sci..

[51] Prgomet I., Gonçalves B., Domínguez-Perles R., Pascual-Seva N., Barros A.I.R.N. (2017). Valorization Challenges to Almond Residues: Phytochemical Composition and Functional Application. Molecules.

[52] Albuquerque B.R., Prieto M.A., Vazquez J.A., Barreiro M.F., Barros L., Ferreira I.C.F.R. (2018). Recovery of bioactive compounds from *Arbutus unedo* L. fruits: Comparative optimization study of maceration/microwave/ultrasound extraction techniques. Food Res. Int..

[53] Bolling B.W., McKay D.L., Blumberg J.B. (2010). The phytochemical composition and antioxidant actions of tree nuts. Asia Pac. J. Clin. Nutr..

[54] Alasalvar C., Bolling B. (2015). Review of nut phytochemicals, fat-soluble bioactives, antioxidant components and health effects. Br. J. Nutr..

[55] Carvalho M., Ferreira P.J., Mendes V.S., Silva R., Pereira J.A., Jerónimo C., Silva B.M. (2010). Human cancer cell antiproliferative and antioxidant activities of *Juglans regia* L.. Food Chem. Toxicol..

[56] Akbari V., Jamei R., Heidari R., Esfahlan J.A. (2012). Antiradical activity of different parts of Walnut (*Juglans regia* L.) fruit as a function of genotype. Food Chem..

[57] Shah U.N., Mir J.I., Ahmed N., Jan S., Fazili K.M. (2018). Bioefficacy potential of different genotypes of walnut *Juglans regia* L.. J. Food Sci. Technol..

[58] Santos A., Barros L., Calhelha R.C., Dueñas M., Carvalho A.M., Santos-Buelga C., Ferreira I.C.F.R. (2013). Leaves and decoction of *Juglans regia* L.: Different performances regarding bioactive compounds and in vitro antioxidant and antitumor effects. Ind. Crop. Prod..

[59] Mocan A., Diuzheva A., Carradori S., Andruch V., Massafra C., Moldovan C., Sisea C., Petzer J.P., Petzer A., Zara S. (2018). Development of novel techniques to extract phenolic compounds from Romanian cultivars of *Prunus domestica* L. and their biological properties. Food Chem. Toxicol..

[60] de la Rosa L.A., Alvarez-Parrilla E., Shahidi F. (2011). Phenolic compounds and antioxidant activity of kernels and shells of Mexican pecan (*Carya illinoinensis*). J. Agric. Food Chem..

[61] Lin J.T., Liu S.C., Hu C.C., Shyu Y.S., Hsu C.Y., Yang D.J. (2016). Effects of roasting temperature and duration on fatty acid composition, phenolic composition, Maillard reaction degree and antioxidant attribute of almond (*Prunus dulcis*) kernel. Food Chem..

[62] Lainas K., Alasalvar C., Bolling B.W. (2016). Effects of roasting on proanthocyanidin contents of Turkish Tombul hazelnut and its skin. J. Funct. Foods.

[63] Chang S.K., Alasalvar C., Bolling B.W., Shahidi F. (2016). Nuts and their co-products: The impact of processing (roasting) on phenolics, bioavailability, and health benefits-A comprehensive review. J. Funct. Foods.

[64] Payne M.J., Hurst W.J., Miller K.B., Rank C., Stuart D.A. (2010). Impact of fermentation, drying, roasting, and dutch processing on epicatechin and catechin content of cacao beans and cocoa ingredients. J. Agric. Food Chem..

[65] Martinez M.L., Labuckas D.O., Lamarque A.L., Maestri D.M. (2010). Walnut (*Juglans regia* L.): Genetic resources, chemistry, by-products. J. Sci. Food Agric..

[66] Schlörmann W., Birringer M., Böhm V., Löber K., Jahreis G., Lorkowski S., Müller A.K., Schöne F., Glei M. (2015). Influence of roasting conditions on health-related compounds in different nuts. Food Chem..

[67] Figueroa F., Marhuenda J., Zafrlla P., Martínez-Cachá A., Mulero J., Cerdá B. (2016). Total phenolics content, bioavailability and antioxidant capacity of 10 different genotypes of walnut (*Juglans regia* L.). J. Food Nutr. Res..

[68] Alasalvar C., Karamać M., Kosińska A., Rybarczyk A., Shahidi F., Amarowicz R. (2009). Antioxidant activity of hazelnut skin phenolics. J. Agric. Food Chem..

[69] Taş N.G., Gökmen V. (2017). Phenolic compounds in natural and roasted nuts and their skins: A brief review. Curr. Opin. Food Sci..

[70] de la Rosa L.A., Vazquez-Flores A.A., Alvarez-Parrilla E., Rodrigo-García J., Medina-Campos O.N., Ávila-Nava A., González-Reyes S., Pedraza-Chaverri J. (2014). Content of major classes of polyphenolic compounds, antioxidant, antiproliferative, and cell protective activity of pecan crude extracts and their fractions. J. Funct. Foods.

[71] Arcan I., Yemeniciog A. (2009). Antioxidant activity and phenolic content of fresh and dry nuts with or without the seed coat. J. Food Compost. Anal..

[72] Delgado T., Malheiro R., Pereira J.A., Ramalhosa E. (2010). Hazelnut (*Corylus avellana* L.) kernels as a source of antioxidants and their potential in relation to other nuts. Ind. Crop. Prod..

[73] Slatnar A., Mikulic-Petkovsek M., Stampar F., Veberic R., Solar A. (2014). HPLC-MS^n^ identification and quantification of phenolic compounds in hazelnut kernels, oil and bagasse pellets. Food Res. Int..

[74] Panth N., Paudel K.R., Karki R. (2016). Phytochemical profile and biological activity of *Juglans regia*. J. Integr. Med..

[75] Christopoulos M.V., Tsantili E. (2011). Effects of temperature and packaging atmosphere on total antioxidants and colour of walnut (*Juglans regia* L.) kernels during storage. Sci. Hort..

[76] Chen C.Y., Blumberg J.B. (2008). Phytochemical composition of nuts. Asia Pac. J. Clin. Nutr..

[77] Wong W.H., Lee W.X., Ramanan R.N., Tee L.H., Kong K.W., Galanakis C.M., Sun J., Prasad K.N. (2015). Two level half factorial design for the extraction of phenolics, flavonoids and antioxidants recovery from palm kernel by-product. Ind. Crop. Prod..

[78] Fu M., Qu Q., Yang X., Zhang X. (2016). Effect of intermittent oven drying on lipid oxidation, fatty acids composition and antioxidant activities of walnut. LWT-Food Sci. Technol..

[79] Zhang Z., Liao L., Moore J., Wu T., Wang Z. (2009). Antioxidant phenolic compounds from walnut kernels (*Juglans regia* L.). Food Chem..

[80] Hooper L., Kay C., Abdelhamid A., Kroon P.A., Cohn J.S., Rimm E.B., Cassidy A. (2012). Effects of chocolate, cocoa, and flavan-3-ols on cardiovascular health: A systematic review and meta-analysis of randomized trials. Am. J. Clin. Nutr..

[81] Ellinger S., Reusch A., Stehle P., Helfrich H.P. (2012). Epicatechin ingested via cocoa products reduces blood pressure in humans: A nonlinear regression model with a Bayesian approach. Am. J. Clin. Nutr..

[82] Khalesi S., Sun J., Buys N., Jamshidi A., Nikbakht-Nasrabadi E., Khosravi-Boroujeni H. (2014). Green tea catechins and blood pressure: A systematic review and meta-analysis of randomised controlled trials. Eur. J. Nutr..

[83] Pei J., Chen A., Zhao L., Cao F., Ding G., Xiao W. (2017). One-pot synthesis of hyperoside by a three-enzyme cascade using a UDP-galactose regeneration system. J. Agric. Food Chem..

[84] Biswas R., Mukherjee P.K., Chaudhary S.K. (2016). Tyrosinase inhibition kinetic studies of standardized extract of *Berberis aristata*. Nat. Prod. Res..

[85] Tan X., Song Y.H., Park C., Lee K.W., Kim J.Y., Kim D.W., Kim K.D., Lee K.W., Curtis-Long M.J., Park K.H. (2016). Highly potent tyrosinase inhibitor, neorauflavane from *Campylotropis hirtella* and inhibitory mechanism with molecular docking. Bioorg. Med. Chem..

[86] Malik W., Ahmed D., Izhar S. (2017). Tyrosinase Inhibitory Activities of *Carissa opaca* Stapf ex Haines Roots Extracts and Their Phytochemical Analysis. Pharmacogn. Mag..

[87] Uysal S., Zengin G., Aktumsek A., Karatas S. (2016). Chemical and biological approaches on nine fruit tree leaves collected from the Mediterranean region of Turkey. J. Funct. Foods.

[88] Quispe Y.N., Hwang S.H., Wang Z., Lim S.S. (2017). Screening of Peruvian medicinal plants for tyrosinase inhibitory properties: Identification of tyrosinase inhibitors in *Hypericum laricifolium* Juss. Molecules.

[89] Savran A., Zengin G., Aktumsek A., Mocan A., Glamoćlija J., Ćirić A., Soković M. (2016). Phenolic compounds and biological effects of edible *Rumex scutatus* and *Pseudosempervivum sempervivum*: Potential sources of natural agents with health benefits. Food Funct..

[90] Mocan A., Moldovan C., Zengin G., Bender O., Locatelli M., Simirgiotis M., Atalay A., Vodnar D.C., Rohn S., Crișan G. (2018). UHPLC-QTOF-MS analysis of bioactive constituents from two Romanian Goji (*Lycium barbarum* L.) berries cultivars and their antioxidant, enzyme inhibitory, and real-time cytotoxicological evaluation. Food Chem. Toxicol..

